# Sexuality, mother-child health and infectious diseases

**DOI:** 10.4314/ahs.v17i3.1

**Published:** 2017-09

**Authors:** James K Tumwine

Welcome to this September 2017 issue of *African Health Sciences*. We have three main themes in the issue: sexuality/reproductive health; infectious diseases and health system issues.

## Sexuality, mother and child health

The main article is on the efficacy of traditional Chinese medicine for the treatment of premature ejaculation. The authors found that traditional Chinese medicine spray (TCMS) was safe and effective for the treatment of premature ejaculation.^1^

Nigerian authors on the other hand report on the prevalence and factors associated with early sexual debut among secondary school students in South West Nigeria.^2^ The next two papers from Iran are also on sexuality. Amiri and colleagues describe female sexual outcomes in primiparous women after vaginal delivery and caesarian section;^3^ while the next paper is on the diagnosis and treatment of unconsummated marriage in an Iranian couple.^4^

Ethiopian workers present a logistic regression model with complex sampling design of unmet need for family planning among all women aged (15–49) years in Ethiopia.^5^ On the other hand, Algerian workers report on the contribution of IgG avidity and PCR for the early diagnosis of toxoplasmosis in pregnant women.^6^ South African workers report on compliance to the consumption of iron and folate supplements by pregnant women in a local community in Mafeking.^7^ The section ends with determinants of obstetric fistula, that is so rampant in sub Saharan Africa.^8^

Intimate partner violence (IPV) is a serious issue in sub Saharan Africa. Ethiopian workers found that almost 1 in 5 pregnant women experienced IPV, which was associated with low birth weight.^9^ Still, with newborn and child health theme, we bring you determinants of pre-lacteal feeding practices based on the 2013 Nigeria demographic and health survey data.^10^ We end this first theme with an assessment of policymakers' individual and organizational capacity to acquire, assess, adapt and apply research evidence for maternal and child health policy making.^11^

## Infectious diseases

The next papers are on infectious diseases. Hence Sharew and others report on HIV seroprevalence trend among blood donors in Ethiopia.^12^ We have work on acute systematic inflammatory response after cardiac surgery in patients infected with HIV virus.^13^ Still on the HIV theme, Ugandanauthors report results of a study on HIV counselling and testing among clients presenting at a market HIV clinic in Kampala.^14^ Next we have a paper on adherence to ART,^15^ decentralization of ART management,^16^ and influence of faith based organizations on HIV prevention.^17^ One paper documents HBV DNA gene polymorphisms prediction of chronic HBV infection in India.^18^

Keeping with the infectious disease theme, we have 2 papers on TB: prevalence of DM in newly diagnosed PTB patients in Beira Mozambique,^19^ anti TB activity of extracts of herbal medicine in South Africa^20^, and antibacterial activity of an indigenous plant(*Grewiaflava*) in South Africa.^21^

Next bacteriology. Here we have issues on multiple drug resistant *Staphylococcus aureus* in Nigeria^22^ and repurposing metformin as a quorum sensing inhibitor of *Pseudomonas aeruginosa*.^23^ Bai and colleagues report meteorological factors associated with *meningococcal* meningitis^24^ and Nworu and others share their work on anti-plasmodial activities of *Rubiaceae* in Nigeria.^25^

## Mental health

We have a paper on common mental disorders among medical students in Jimma University, SouthWest Ethiopia,^26^ followed by one on the pattern and outcome of EEGs in the management of neuro-psychiatric disorders.^27^ This is followed by a paper on the burden and factors associated with post-stroke depression in East Central Nigeria.^28^

The section ends with a paper on prevalence and correlates of anxiety and depression among carers of cancer patients in the Uganda Cancer Institute.^29^

## Others

Finally there is a diverse paper on visual impairment among children attending the National Referral Hospital Eye Clinic, Uganda.^30^

This is followed by two related papers. One is on factors associated with tobacco smoking behavior among long distance drivers in Lagos,^31^ while the other reports that cigarette smoke condensate attenuates phobol ester-mediated neutrophil extracellular trap formation.^32^

Dental caries are a serious menace in our lives as one visit to the dentist will demonstrate. Hence Lawal and others report treatment needs of women in Nigeria.^33^

Methodological challenges in a study on falls of the elderly in Cape Town, South Africadeserve attention^34^; while Kyei and others explore the onset and duration of cycloplegic action of 1% cyclopentolaate-1% tropicamide combination.^35^

We end this treatise with two diverse but equally fascinating topics: iron transport from ferrous glycinate liposomes^36^ and client satisfaction in a faith based health network in Uganda.^37^
